# Control of the olive fruit fly using genetics-enhanced sterile insect technique

**DOI:** 10.1186/1741-7007-10-51

**Published:** 2012-06-19

**Authors:** Thomas Ant, Martha Koukidou, Polychronis Rempoulakis, Hong-Fei Gong, Aris Economopoulos, John Vontas, Luke Alphey

**Affiliations:** 1 Oxitec Limited, 71 Milton Park, Oxford OX14 4RX, UK; 2 Department of Zoology, University of Oxford, South Parks Road, Oxford OX1 3PS, UK; 3 Faculty of Biotechnology and Applied Biology, Department of Biology, University of Crete, Heraklion, Crete, Greece

**Keywords:** olive fly, Bactrocera oleae, sterile insect technique, SIT, release of insects carrying a dominant lethal, RIDL, autocidal control, insect transgenics

## Abstract

**Background:**

The olive fruit fly, *Bactrocera oleae*, is the major arthropod pest of commercial olive production, causing extensive damage to olive crops worldwide. Current control techniques rely on spraying of chemical insecticides. The sterile insect technique (SIT) presents an alternative, environmentally friendly and species-specific method of population control. Although SIT has been very successful against other tephritid pests, previous SIT trials on olive fly have produced disappointing results. Key problems included altered diurnal mating rhythms of the laboratory-reared insects, resulting in asynchronous mating activity between the wild and released sterile populations, and low competitiveness of the radiation-sterilised mass-reared flies. Consequently, the production of competitive, male-only release cohorts is considered an essential prerequisite for successful olive fly SIT.

**Results:**

We developed a set of conditional female-lethal strains of olive fly (named Release of Insects carrying a Dominant Lethal; RIDL^®^), providing highly penetrant female-specific lethality, dominant fluorescent marking, and genetic sterility. We found that males of the lead strain, OX3097D-Bol, 1) are strongly sexually competitive with wild olive flies, 2) display synchronous mating activity with wild females, and 3) induce appropriate refractoriness to wild female re-mating. Furthermore, we showed, through a large proof-of-principle experiment, that weekly releases of OX3097D-Bol males into stable populations of caged wild-type olive fly could cause rapid population collapse and eventual eradication.

**Conclusions:**

The observed mating characteristics strongly suggest that an approach based on the release of OX3097D-Bol males will overcome the key difficulties encountered in previous olive fly SIT attempts. Although field confirmation is required, the proof-of-principle suppression and elimination of caged wild-type olive fly populations through OX3097D-Bol male releases provides evidence for the female-specific RIDL approach as a viable method of olive fly control. We conclude that the promising characteristics of OX3097D-Bol may finally enable effective SIT-based control of the olive fly.

## Background

The olive fly, *Bactrocera oleae *(Rossi) (Diptera: Tephritidae), is the major insect pest of olives. Females typically lay a single egg per olive [1], injected into the fruit through the female's ovipositor. The developing larva tunnels through the olive, feeding on the fleshy mesocarp. Heavy olive fly infestation can reduce the quality and therefore the value of the olive oil by up to 80%, and cause the rejection of entire harvests of table olives [2]. Control currently relies overwhelmingly on the use of chemical insecticides, and because of the high economic and environmental costs of chemical control, together with the appearance of insecticide-resistant populations [3], there is an urgent need for improved control methods.

The sterile insect technique (SIT) is an environmentally friendly and species-specific method of pest control based on the release of large numbers of sterilised insects [4]. Competition for mating between wild and sterile males results in a decrease in the number of fertile matings, and a decline in the overall population size. SIT has been successfully implemented against various pest insect species including several Tephritidae. However, despite decades of research aimed at developing an olive fly SIT programme using radiation-sterilised flies [5], the consistently poor results led to eventual abandonment of the trials. Key issues included low quality of the radiation-sterilised mass-reared flies, economical production of sufficient numbers of sterilised flies, and assortative mating of released and wild populations because of different preferred mating times [5-7]. Laboratory-reared wild-type flies were found to mate several hours earlier than wild flies [7] (presumably due to differential selective pressures in the artificial laboratory-rearing environment). The proposed solution was male-only release [6]. Male-only release using a genetic sexing strain has also been shown to give a threefold to fivefold improvement in the performance of released radiation-sterilised *Ceratitis capitata *(Medfly) males used for SIT [8].

The RIDL^® ^(Release of Insects carrying a Dominant Lethal) system is a transgene-based derivative of SIT [9-14], one version of which involves the mass release of insects carrying a female-specific lethal transgene (fsRIDL) [15-17]. Encouraged by work advancing olive fly mass rearing by the International Atomic Energy Agency (IAEA) and others, we set out to develop fsRIDL strains of olive fly to overcome the remaining historic limitations of olive fly SIT. We therefore transformed the olive fly with construct OX3097, an fsRIDL system that had previously provided suitable fsRIDL strains for Medfly, incorporating genetic sexing, genetic sterilisation, and a heritable fluorescent marker [15].

We report the development of the first transgenic control strains for the olive fly. The lead strain, OX3097D-Bol, provides highly penetrant, dominant, female-specific lethality when reared in the absence of a transgene repressor. Using a series of behavioral studies, we found that OX3097D-Bol males show fully synchronous and strongly competitive mating behavior with wild olive flies, and display no reduction in their ability to induce refractoriness to wild female re-mating. This is a more stringent test than using laboratory wild-type flies, which may have co-adapted to laboratory rearing conditions. Furthermore, we describe results from experiments showing the ability of periodic releases of OX3097D-Bol males to suppress large caged wild-type olive fly populations.

## Results

### Olive fly transformation with OX3097 and phenotypic analysis

**Figure 1:**
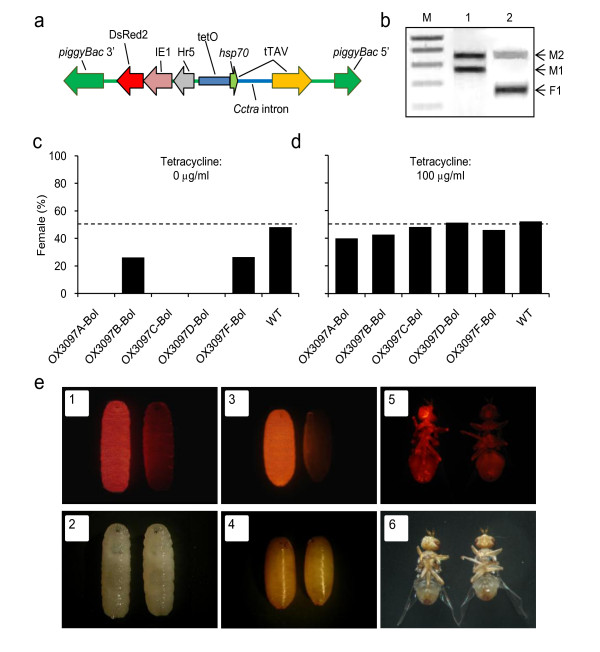
**The OX3097 transposon and induced phenotypes in olive fly**. (**A**) Diagrammatic representation of the OX3097 transposon. OX3097 comprises a fluorescent marker (hr5-IE1-DsRed2), and the female-specific tTAV expression system (tetO-*Dmhsp70 *minimal promoter - Cctra:tTAV) [15]. Sex-specific alternative splicing of the *Cctra *intron leads to production of tTAV and the initiation of a lethal tTAV positive-feedback loop in females only [14,15,34]. (**b**) Products of alternative splicing of *Cctra*:tTAV in (lane 1) male and (lane 2) female OX3097D-Bol olive flies. Three splice variants were detected, corresponding to Cctra transcripts M1, M2 and F1 [18] (identity confirmed by sequencing). Only females produce the F1 splice variant, corresponding to the reconstitution of the tTAV open reading frame and leading to production of functional tTAV. Lane M shows DNA size standards: 200-1,000 bp in 200-bp increments (Eurogentec Smartladder). (**C**) Penetrance and (**D**) tetracycline repressibility of female lethality in five OX3097 olive fly lines. Strains OX3097A-D-Bol &F-Bol are five insertion lines of OX3097 in olive fly. Penetrance and repressibility of female-specific lethality was assessed by crossing heterozygous males of each strain to virgin wild-type (WT) females, and collecting eggs on filter paper saturated with water containing either 0 μg/ml tetracycline or 100 μg/ml tetracycline. The sex ratio of adult progeny expressing the DsRed2 fluorescent marker is shown for each strain compared with wild-type (WT) progeny. Lines A, C and D showed fully penetrant female-specific lethality when reared in the absence of tetracycline (off-tet); that is, they produced no female progeny off-tet in this assay. In lines C and D, female-specific lethality was also efficiently repressed on-tet. (**E**) Fluorescence microscopy allows discrimination of OX3097D-Bol from wild type at larval, pupal, and adult stages. Photomicrographs of OX3097D-Bol and wild-type olive flies under (upper panels) fluorescence and (lower panels) bright-field illumination. Each panel shows OX3097D-Bol to the left and wild-type to the right: OX3097D-Bol and wild-type (1,2) larvae, (3,4) pupae, and (5,6) adults are shown. Expression of DsRed2 is clearly visible all over the OX3097D-Bol larvae and pupae, and in areas of less opaque cuticle (for example, the labellum, upper thorax, leg joints, and anus) of OX3097D-Bol adults.

Six transgenic lines were generated carrying the OX3097 transgene (Figure 1A). Of the five lines with single insertions, two showed female-specific lethality that was fully penetrant and efficiently repressed by dietary tetracycline (Figure 1C, D). Of these, line OX3097D-Bol (Figure 1E), which also showed the brightest expression of the DsRed2 fluorescent marker, was selected for further analysis. Consistent with previous observations of the OX3097 construct in Medfly [15] and Mexfly (Stainton *et al., *unpublished data), female lethality occurred at the early pupal stage. Analysis of transgene-derived transcripts indicated that the alternative splicing pattern of the *Cctra *intron in OX3097D-Bol olive fly is equivalent to that in its native context [15,18,19] (Figure 1B). The OX3097D-Bol strain was selected for further development, and made homozygous for the transgene.

### Mating tests with OX3097D-Bol and wild olive flies

**Figure 2:**
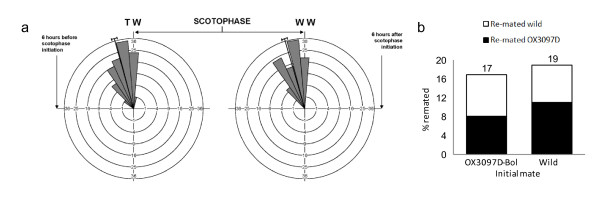
**Mating initiation times and re-mating propensity of OX3097D-Bol with wild olive flies**. (**A**) Copulation initiation times were similar for OX3097D-Bol males and wild males. Copulation initiation times were recorded for all mating pairs; each pair contained a wild female and either an OX3097D-Bol male (left circle, n = 216) or a wild male (right circle, n = 161). Scotophase is the dark phase of a light/dark cycle. Each 'wedge' on the circular graphic represents a time-interval of 45 minutes; the radial length of the wedge indicates the proportion of total matings of that type that occurred in each time segment. Mean copulation initiation time for wild females and either OX3097D-Bol or wild males was 63 and 66 minutes before scotophase respectively. Peak mating activity times were not significantly different between the two types of male (*P *= 0.45, degrees of freedom (d.f.) = 1, circular statistics *F*-test). (**B**) Genotype of first mate (OX3097D-Bol or wild) did not affect female re-mating frequency or genotype of second mate. Of 188 females initially mated to OX3097D-Bol males 32 (17%) re-mated, of which 17 (9%) re-mated to wild males (open portion of left bar), and 13 (8%) to OX3097D-Bol males (solid portion of left bar). Of 296 females initially mated to wild males 55 (19%) re-mated, of which 23 (8%) re-mated to wild males (open portion of right bar) and 32 (11%) to OX3097D-Bol males (solid portion of left bar). Re-mating propensity of wild females initially mated with either an OX3097D-Bol male or a wild male were not significantly different (*P *= 0.7, d.f. = 1, χ^2 ^test). Furthermore, the re-mating preference of the wild females was not found to differ significantly depending on first-mate choice (*P *= 0.38, d.f. = 1, χ^2 ^test).

Caged mating competitiveness tests challenging homozygous OX3097D-Bol males to compete with wild males for copulations with wild females were performed based on established guidelines [20], using wild olive flies from infested olives collected in Crete. In total, 15 experiments were performed, each with 50 OX3097D-Bol males, 50 wild males, and 50 wild females, thus more than 400 total couples were assessed. Wild males outperformed OX3097D-Bol males, gaining an average of 56% of total mates (*P *= 0.01, degrees of freedom (d.f.) = 1, likelihood ratio test for goodness of fit, n = 406). Nonetheless, this near-equal outcome far exceeds established thresholds for SIT [20]. Copulation initiation times were also recorded (Figure 2A). No significant difference was seen in mating initiation times for each type of male (*P *= 0.44, d.f. = 1, circular statistics *F*-test), suggesting synchronicity in mating activity between OX3097D-Bol males and wild flies.

Olive fly females typically only mate once [21]; males transfer more sperm than the female needs to fertilise all her eggs, and mating induces long-lasting refractoriness to re-mating, although if the females are held in close proximity to males some re-mating occurs. For Medfly, radiation sterilisation not only reduces the ability of male flies to compete for mates but also increases the likelihood that mated females will subsequently re-mate; that is, radiation reduces the ability of the males to induce refractoriness to re-mating in females [22]. Furthermore, the second mate taken is also preferentially wild rather than sterile. These effects of reduced male mating success, increased female re-mating, and preferential mating to wild (fertile) males on re-mating each reduce the efficiency of SIT. We therefore investigated the effect of first-male genotype on wild female re-mating propensity and second-mate genotype. Wild females who had initially mated either an OX3097D-Bol male (n = 188) or a wild male (n = 296) were caged with and allowed to mate with equal proportions of OX3097D-Bol and wild males over 15 days. No significant differences in re-mating rate (*P *= 0.7, d.f. = 1, χ^2 ^test), or second-mate genotype were seen (*P *= 0.38, d.f. = 1, χ^2 ^test) (Figure 2B).

### Elimination of caged olive fly populations

**Figure 3:**
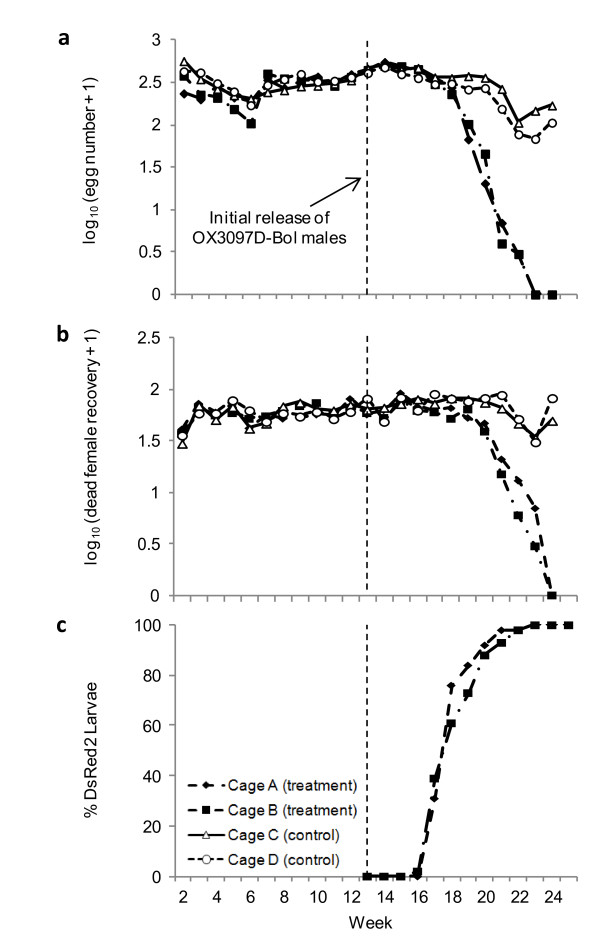
**Population elimination by periodic release of OX3097D-Bol males**. (**A**) The average daily egg production for each cage. Weeks 1 to 12 was the population stabilization period with 250 pupae added in the first week, and 200 pupae added to each cage per week thereafter. From week 13, 1,600 OX3097D-Bol pupae were added weekly into cages A and B. After week 13, weekly pupal return to the treatment cages was made proportional to the weekly egg production in the cage relative to the control cages. From 5 weeks after initiation of RIDL introductions, egg production in each treatment cage was consistently lower than in either control cage; the difference increased until eventual extinction of the wild-type population in both treatment cages by week 24 (12 weeks after the first RIDL release). Extinction was defined as 2 weeks of zero egg production. Egg numbers in control cages remained relatively stable. (**B**) Dead flies were removed from the cages weekly, and the numbers of dead females are shown. From 7 weeks after the initiation of RIDL release, increasingly fewer such females were recovered from the treatment cages than from the control cages. (**C**) Frequency of DsRed2 in treatment cages. Larvae selected for return were screened for presence of DsRed2 marker by fluorescence microscopy before being returned to the treatment cage (see Methods). The proportion of returning pupae carrying the OX3097D-Bol transgene reached 100% in both treatment cages by week 23 (10 weeks post-RIDL release). Olive fly females typically mate only once [21] (Figure 2B). Females start to lay eggs approximately 2 days after mating, and lay most of their eggs within the next 10 days. Egg to pupa development time was approximately 12 days. These pupae therefore indicate female mating choice of approximately 3 weeks before each measurement.

We tested the ability of periodic release of OX3097D-Bol males to suppress target populations of wild-type olive fly, as published previously [17]. Wild-type populations totaling approximately 400 male and female olive flies were established in each of four large (8 m^3^) cages in a greenhouse at the University of Crete. At week 13 from first establishment, approximately 1,600 OX3097D-Bol male pupae were introduced per week. Population density was monitored by measuring egg production (Figure 3A) and female mortality (Figure 3B). To track mating outcomes, pupae taken from the cage were scored for fluorescence (Figure 3C). Fluorescent pupae were detected at three weeks post-first release (PR) and the proportion increased rapidly thereafter to 100% by week 10 PR. Death of fluorescent (transgenic) female pupae as a result of expression of the lethal phenotype would reduce the number of egg-laying adult females; based on the fluorescence data, no wild-type females emerged (eclosed) in the treatment cages from week 10 PR. Egg production was lower in the treatment than in the control cages in week 6 PR, and declined rapidly thereafter. The numbers of dead females recovered from treatment cages started to decrease from around week 6 to 7 PR, presumably due to a decline in overall female numbers. Defining extinction as 2 weeks of zero egg production [17], both treatment cage populations were extinct by week 12 PR.

## Discussion

An effective non-chemical approach to olive fly management is highly desirable, and SIT could provide this if specific technical issues can be overcome. Considerable progress has been made by others in respect of olive fly mass-rearing and release, and we set out to overcome the key remaining issues through genetics.

The use of genetic technology in food production has been controversial in some countries, notably within the European Union (EU). It is possible that the use of genetically modified arthropods to protect the crop in a sustainable way, which leaves little or no residue in the food product (here olives or olive oil) might be more acceptable in the EU than food products that are produced directly from genetically modified organisms. For comparison, the Standing Committee on the Food Chain and Animal Health stated that foods and feeds produced by fermentation with genetically modified microorganisms that are not present in the final product are "excluded from the scope of regulations" [23]. Additionally, it is recognized in EU legislation that the presence of technically unavoidable traces of genetically modified organisms in food and feed products should not trigger labeling requirements (Regulation 1830/2003/EC), as long as appropriate steps to avoid the presence of the materials have been taken. This is also analogous to the situation regarding the presence of wild-type insect parts as contaminants in food products [24].

Using modern genetic engineering techniques, we have developed OX3097D-Bol, an fsRIDL olive fly strain that incorporates engineered phenotypes, directly addressing problems encountered in previous unsuccessful olive fly SIT trials [5]. OX3097D-Bol is able to provide 100% male-only release generations when reared in the absence of a dietary transgene repressor. Genetic sexing encourages random mating between the wild and the released sterile populations, providing established performance benefits [8]. Female lethality renders redundant the requirement of performance-reducing and costly sterilisation using ionising radiation. Furthermore, the dominant fluorescent marker has the potential to improve field monitoring, and the requirement of tetracycline for female development mitigates risks of accidentally released transgenic populations establishing in the wild. Introgression of susceptibility alleles through the female line provides a resistance management option for other control methods used with fsRIDL in an integrated pest-management programme [25-27].

## Conclusions

The good mating characteristics of OX3097D-Bol males seen in this study, including high sexual competitiveness, photo-period compatibility, and efficient induction of re-mating refractoriness in wild females, suggest a strong potential for application in a SIT control programme. Although the results presented here need field confirmation, the proof-of-principle suppression and elimination of caged wild-type olive fly populations through OX3097D-Bol male releases provides evidence for the fsRIDL approach as a viable method for olive fly control. Independently, significant advances have been achieved in olive fly mass-rearing [28,29] and release [30] techniques. We therefore propose that the development of OX3097D-Bol will help form the basis of more effective and more sustainable control of this ancient and destructive olive pest.

## Methods

### Olive fly strains, rearing, and transformation

Olive fly rearing was carried out using standard methods. Approximately 4,500 pre-blastoderm stage olive fly embryos of the Democritus laboratory (Greece) *B. oleae *strain were micro-injected with the OX3097 plasmid and *piggyBac *mRNA. as described by Koukidou *et al. *[31]. This resulted in 138 surviving G_0 _adults. After back-crossing pools of five male survivors with ten wild-type females, and five female survivors with five wild-type males, six OX3097 lines were isolated (transformation efficiency ~4%). Transgenics were outcrossed for five generations to the Argov wild-type strain (Israel), and these outcrossed Argov derivatives were used for all experiments. The Argov strain was derived from field collections of wild male olive fly in Israel, outcrossed to Democritus females. To develop a homozygous strain, a pool of homozygous and heterozygous OX3097D-Bol adults was generated by crossing OX3097D-Bol heterozygotes. DNA from these parents was analysed by PCR using primers (5'-CCTGCGTTTGGAGATGACGAAATC-3' and 5'-CTTACATATAGAGCAGTGCGCTCACATG-3') that anneal to genomic sites flanking the insertion site producing a WT (no insertion) amplicon were discarded. Homozygotes were thereby identified, and a homozygous line was developed from 15 female and 13 male founder flies. The key phenotypic properties identified in OX3097D-Bol heterozygotes (Figure 1, 2) were also found in the homozygous line.

### Reverse transcriptase PCR

Male and female tTAV [14,34] transcripts were amplified by reverse transcriptase (RT)-PCR using cDNA synthesized using a commercial kit (Superscript II; Life Technologies, Grand Island, NY, USA) on RNA extracts prepared using TRI reagent (Life Technologies). The major RT-PCR products were sequenced (GATC Biotech, Konstanz, Germany) and analysed using VectorNTi (Life Technologies).

### Mate competition and re-mating tests

Mating competitiveness tests were carried out in accordance with the United Nations Food and Agriculture Organization (FAO)/IAEA/United States Department of Agriculture (USDA) guidelines [20] in semi-natural caged conditions (cages were 1.25 m high with a base of 0.25 m^2 ^and contained a large olive branch) in greenhouse facilities at the University of Crete under natural light. Adult male OX3097D-Bol flies were obtained from larvae reared in the absence of tetracycline ("off-tet") at low density (1 larva/0.8 g larval medium). Wild pupae were recovered from infested olives gathered from olive orchards near to the University of Crete. Each mating test used 50 OX3097D-Bol males, 50 wild males, and 50 wild females. Mated males were scored for the presence of the DsRed2 fluorescent marker by epifluorescence microscopy [32,33] (Clontech Laboratories, Inc, Mountain View, CA, USA). OX3097D-Bol mating competition tests were performed in 15 replicates with more than 400 couples assessed in total. Each experiment yielded a mating propensity of greater than 0.2 [20]. Mating cages for the first step of re-mating tests contained either wild females and OX3097D-Bol males or wild females and wild males. Mating couples were removed from cages. Mated females were grouped in accordance with the genotype of the first mate (OX3097D-Bol, n = 188 or wild-type, n = 296) and were transferred the following day to new cages with sufficient fresh wild and OX3097D-Bol males to give a 1:1:1 ratio of mated females, wild males, and OX3097D-Bol males. Cages were checked daily for re-mating events over the following 15 days. Re-mating couples were removed, and the male genotype assessed by epifluorescence microscopy.

### Caged suppression of stable wild-type populations

The protocol for the caged suppression experiment was based on that of Wise de Valdez *et al. *[17]. Stable populations of wild-type (Argov) olive flies were established in four field cages (each 8 m^3 ^and containing an olive tree 1.5 m tall; all the cages were contained within a single large glasshouse) over a 12-week period by introducing a fixed number of pupae to each cage weekly (250 in week 1, and 200 from weeks 2 to 12). To assess egg production rates and to sustain caged olive fly populations, four ceresin-wax cones (combined surface area approximately 550 cm^2^) were added to each cage daily for female oviposition. Pupal additions for the first 4 weeks during population establishment originated from a wild-type stock colony; thereafter, experimental cages were self-sufficient. Eggs were collected from the cages and counted daily. On week 12, the experimental cages were randomly divided into two RIDL-treatment and two no-treatment (control) cages. From week 13 onwards, RIDL-treatment cages received weekly additions of 1,600 OX3097D-Bol male pupae reared off-tet (an initial recruitment rate ratio of approximately 16 OX3097D-Bol males to 1 wild-type male). Once the OX3097D-Bol introductions began, pupal introductions to the RIDL-treatment cages were proportional to the cage's respective rate of egg production, with the control cages providing a coefficient of weekly egg production to pupal return. Numbers of females in a cage were monitored by collecting and counting dead females. The ratio of RIDL heterozygous to wild-type pupae returning to RIDL-treatment cages was monitored by fluorescence microscopy (OX3097D-Bol heterozygous females reared off-tet pupate but fail to eclose). Larvae were screened only after the returning population was separated, to remove the possibility of bias in selecting individual flies for reintroduction to the cage populations.

### Statistical analysis

Comparison of mating competitiveness between OX3097D-Bol and wild males was performed using a likelihood ratio test for goodness of fit with numbers of successfully mated males from both genotypes pooled across experiments, and compared against a theoretical 1:1 genotype ratio expected in random mating. Differences in copulation initiation time between the two possible mating combinations (OX3097D-Bol/wild female and wild male/wild female) were analysed using a circular statistics *F*-test with the statistical software Oriana for Windows (Kovach Computing Services, Anglesey, UK). The Pearson's χ^2 ^test was used to compare numbers of re-mated to non-re-mated females for both initial mating combinations. The same test was used to compare initial and second-mate choice. Unless otherwise stated, all statistical tests were performed using SPSS for Windows (version 10.0; SPSS Inc., Chicago IL USA), with significance set at *P *< 0.05.

## Abbreviations

CcTra intron: *Ceratitis capitata *transformer intron; fsRIDL: female-specific Release of Insects carrying a Dominant Lethal; PR: post-first-release; RIDL: Release of Insects carrying a Dominant Lethal; SIT: Sterile Insect Technique.

## Competing interests

TA, MK, PK, HFG and LA are employees or students of Oxitec Ltd and have employment, stipend and/or equity interest in Oxitec. Oxitec and the University of Oxford have intellectual property related to the subject matter of this paper. All other authors declare no interest.

## Authors' contributions

TA and MK created the transgenic olive fly lines. TA conducted the molecular analysis TA, MK, PR, carried out mating tests and suppression trial. HG, JV and AE provided advice, LA conceived the project, and MK supervised the project. TA and LA wrote the paper. All authors discussed results and commented on the manuscript

## References

[B1] Burrack HJ Fornell AM Connell JH O'Connell NV Phillips PA Vossen PM Zalom FG Intraspecific larval competition in the olive fruit fly (Diptera: tephritidae) Environ Entomol 2009 38 1400 1410 10.1603/022.038.0508 19825295

[B2] Daane KM Johnson MW Olive fruit fly: managing an ancient pest in modern times Annu Rev Entomol 2010 55 151 169 10.1146/annurev.ento.54.110807.090553 19961328

[B3] Vontas J Hernandez-Crespo P Margaritopoulos JT Ortego F Feng H Mathiopoulos KD Hsu J Insecticide resistance in Tephritid flies Pest Biochem and Physiol 2011 100 199 10.1016/j.pestbp.2011.04.004

[B4] Dyck VA Hendrichs J Robinson AS Sterile Insect Technique: Principles and practice in area-wide integrated pest management 2005 Dordrecht, Netherlands: Springer

[B5] Economopoulos AP Avtzis N Zervas G Tsitsipis J Haniotakis G Tsiropoulos G Manoukas A Control of the olive fly, Dacus oleae (Gmelin), by the combined effects of insecticides and release of gamma sterilised insects J Appl Entomol 1977 83 201 215

[B6] Economopoulos AP The olive fly, Bactrocera (Dacus) oleae (Gmelin) (Diptera:Tephritidae): its importance and control; previous SIT research and pilot testing 2001 Austria: International Atomic Energy Agency 44

[B7] Zervas GA Economopoulos AP Mating frequency in caged populations of wild and artificially reared (normal or gamma-sterilized) olive fruit flies, Dacus oleae (Gmelin) (Diptera:Tepritidae) Environ Entomol 1982 11 17 20

[B8] Rendon P McInnis D Lance D Stewart J Medfly (Diptera: Tephritidae) genetic sexing: large-scale field comparison of males-only and bisexual sterile fly releases in Guatemala J Econ Entomol 2004 97 1547 1553 10.1603/0022-0493-97.5.1547 15568342

[B9] Alphey L Nimmo D O'Connell S Alphey N Aksoy S Transgenesis and the management of vector-borne disease 2008 627 Berlin: Springer-Verlag 93 103

[B10] Alphey L Andreasen M Dominant lethality and insect population control Mol Biochem Parasitol 2002 121 2 173 178 10.1016/S0166-6851(02)00040-3 12034450

[B11] Alphey L Re-engineering the sterile insect technique Insect Biochem Mol Biol 2002 32 10 1243 1247 10.1016/S0965-1748(02)00087-5 12225915

[B12] Morrison NI Franz G Koukidou M Miller TA Saccone G Alphey LS Beech CJ Nagaraju J Simmons GS Polito LC Genetic improvements to the sterile insect technique for agricultural pests Asia Pac J Mol Bio 2010 18 275 295

[B13] Thomas DD Donnelly CA Wood RJ Alphey LS Insect population control using a dominant, repressible, lethal genetic system Science 2000 287 5462 2474 2476 10.1126/science.287.5462.2474 10741964

[B14] Gong P Epton MJ Fu G Scaife S Hiscox A Condon KC Condon GC Morrison NI Kelly DW Dafa'alla T Coleman PG Alphey L A dominant lethal genetic system for autocidal control of the Mediterranean fruit fly Nat Biotechnol 2005 23 4 453 456 10.1038/nbt1071 15750586

[B15] Fu G Condon KC Epton MJ Gong P Jin L Condon GC Morrison NI Dafa'alla TH Alphey L Female-specific insect lethality engineered using alternative splicing Nat Biotechnol 2007 25 3 353 357 10.1038/nbt1283 17322873

[B16] Fu G Lees RS Nimmo D Aw D Jin L Gray P Berendonk TU White-Cooper H Scaife S Kim Phuc H Marinotti O Jasinskiene N James AA Alphey L Female-specific flightless phenotype for mosquito control Proc Natl Acad Sci USA 2010 107 12 4550 4554 20176967 10.1073/pnas.1000251107 PMC2826341

[B17] Wise de Valdez MR Nimmo D Betz J Gong HF James AA Alphey L Black WC4th Genetic elimination of dengue vector mosquitoes Proc Natl Acad Sci USA 2011 108 12 4772 4775 10.1073/pnas.1019295108 21383140 PMC3064365

[B18] Pane A Salvemini M Delli Bovi P Polito C Saccone G The transformer gene in Ceratitis capitata provides a genetic basis for selecting and remembering the sexual fate Development 2002 129 15 3715 3725 12117820 10.1242/dev.129.15.3715

[B19] Dafa'alla T Fu G Alphey L Use of a regulatory mechanism of sex determination in pest insect control J Genet 2010 89 3 301 305 10.1007/s12041-010-0041-y 20876996

[B20] FAO/IAEA/USDA Product quality control and shipping procedures for sterile mass-reared tephritid fruit flies 2003 Vienna, Austria: International Atomic Energy Agency

[B21] Tzanakakis KE Tsitsipis JA Economopoulos AP Frequency of mating in females of olive fruit fly under laboratory conditions J Econ Entomol 1968 61 1309 1312

[B22] Kraaijeveld K Chapman T Effects of male sterility on female remating in the Mediterranean fruit fly, Ceratitis capitata Proc Biol Sci 2004 271 209 11 10.1098/rsbl.2003.0116 PMC1810007 15252986

[B23] European Union Report from the Commission to the Council and the European Parliament on the implementation of Regulation (EC) No 1829/2003 of the European Parliament and of the Council on genetically modified food and feed http://eur-lex.europa.eu/LexUriServ/LexUriServ.do?uri=CELEX:52006DC0626:EN:HTML

[B24] European Union Commission Implementing Regulation (EU) No 543/2011 of 7 June 2011 laying down detailed rules for the application of Council Regulation (EC) No 1234/2007 in respect of the fruit and vegetables and processed fruit and vegetables sectors Official Journal of the European Union 2011 543/2011

[B25] Alphey N Bonsall MB Alphey L Modelling resistance to genetic control of insects J Theor Biol 2011 270 1 42 55 10.1016/j.jtbi.2010.11.016 21075122

[B26] Alphey N Bonsall MB Alphey L Combining pest control and resistance management: synergy of engineered insects with Bt crops J Econ Entomol 2009 102 2 717 732 10.1603/029.102.0233 19449654

[B27] Alphey N Coleman PG Donnelly CA Alphey L Managing insecticide resistance by mass release of engineered insects J Econ Entomol 2007 100 5 1642 1649 10.1603/0022-0493(2007)100[1642:MIRBMR]2.0.CO;2 17972643

[B28] Dimou I Rempoulakis P Economopoulos AP Olive fruit fly [Bactrocera (Dacus) oleae (Rossi) (Diptera:Tephritidae)] adult rearing diet without antibiotic J Appl Entomol 2009 134 72 79

[B29] Estes AM Nestel D Belcari A Jessup A Rempoulakis P Economopoulos AP A basis for the renewal of sterile insect technique for the olive fly, Bactrocera oleae (Rossi) J Appl Entomol 2011 136 1 16

[B30] Rempoulakis P Nestel D Dispersal ability of marked, irradiated olive fruit flies [Bactrocera oleae (Rossi) (Diptera: Tephritidae)] in arid regions J Appl Entomol 2012 136 171 180 10.1111/j.1439-0418.2011.01623.x

[B31] Koukidou M Klinakis A Reboulakis C Zagoraiou L Tavernarakis N Livadaras I Economopoulos A Savakis C Germ line transformation of the olive fly Bactrocera oleae using a versatile transgenesis marker Insect Mol Biol 2006 15 1 95 103 10.1111/j.1365-2583.2006.00613.x 16469073

[B32] Lukyanov KA Fradkov AF Gurskaya NG Matz MV Labas YA Savitsky AP Markelov ML Zaraisky AG Zhao X Fang Y Tan W Lukyanov SA Natural animal coloration can Be determined by a nonfluorescent green fluorescent protein homolog J Biol Chem 2000 275 34 25879 25882 10.1074/jbc.C000338200 10852900

[B33] Matz MV Fradkov AF Labas YA Savitsky AP Zaraisky AG Markelov ML Lukyanov SA Fluorescent proteins from nonbioluminescent Anthozoa species Nat Biotechnol 1999 17 10 969 973 10.1038/13657 10504696

[B34] Phuc HK Andreasen MH Burton RS Vass C Epton MJ Pape G Fu G Condon KC Scaife S Donnelly CA Coleman PG White-Cooper H Alphey L Late-acting dominant lethal genetic systems and mosquito control BMC Biol 2007 5 11 10.1186/1741-7007-5-11 17374148 PMC1865532

